# Minimizing Lymphatic Morbidity: Incidence of Lower Extremity Lymphedema After vNOTES-Assisted Sentinel Node Mapping in Endometrial Cancer

**DOI:** 10.3390/curroncol33040208

**Published:** 2026-04-07

**Authors:** Duygu Kurtulus, Kevser Arkan, Ali Deniz Erkmen, Gul Cavusoglu Colak, Sedat Akgol, Behzat Can

**Affiliations:** 1 Department of Physical Therapy and Rehabilitation, Faculty of Medicine, Memorial Atasehir Hospital, T.C. Üsküdar University, 34662 Istanbul, Turkey; 2 Department of Obstetrics and Gynecology, Division of Gynecologic Oncology, Diyarbakir Gazi Yasargil Research and Training Hospital, 21010 Diyarbakir, Turkey; kevser.toprak1989@gmail.com (K.A.); drsedatakgol@gmail.com (S.A.); 3 Department of Obstetrics and Gynecology, Diyarbakir Gazi Yasargil Research and Training Hospital, 21010 Diyarbakir, Turkey; 4 Department of Obstetrics and Gynecology, Division of Gynecologic Oncology, Bower Hospital, 21100 Diyarbakır, Turkey; drbehzatcan@gmail.com

**Keywords:** endometrial cancer, vNOTES, sentinel lymph node mapping, minimally invasive surgery, postoperative lymphedema, surgical outcomes

## Abstract

**Background**: Endometrial cancer is the most common gynecologic malignancy in developed countries. Sentinel lymph node (SLN) mapping has emerged as a less invasive alternative to systematic lymphadenectomy and is increasingly incorporated into surgical staging algorithms. Vaginal natural orifice transluminal endoscopic surgery (vNOTES) provides transvaginal access to the retroperitoneum and may facilitate SLN mapping while potentially reducing postoperative morbidity, including lower extremity lymphedema (LEL). Objective: This study aimed to evaluate the feasibility of vNOTES hysterectomy with bilateral salpingo-oophorectomy (BSO) and retroperitoneal SLN mapping and to report early postoperative lymphedema outcomes in patients with newly diagnosed endometrial cancer. **Methods**: This retrospective cohort study included 113 patients who underwent vNOTES-assisted hysterectomy with BSO and SLN mapping using methylene blue dye at a tertiary referral center between January 2022 and January 2023. Lymphedema was evaluated using the Gynecologic Cancer Lymphedema Questionnaire at 6 and 12 months postoperatively, supported by clinical examination. Descriptive statistical analyses were performed to summarize clinical characteristics and symptom profiles. **Results**: The mean patient age was 55.0 ± 10.5 years and the mean BMI was 30.94 ± 2.54 kg/m^2^. Endometrioid adenocarcinoma was the most common histological subtype (75.5%), and most tumors were grade 1 (57.1%). SLN mapping was successful in 102 of 113 patients (overall detection rate 90.3%), with bilateral detection in 79.6% and unilateral detection in 10.6% of cases. Limb swelling was reported in 4.1% of patients, while only one patient (1.0%) met the criteria for self-reported mild lymphedema. No clinical signs of inguinal lymphedema were detected. **Conclusions**: vNOTES hysterectomy combined with retroperitoneal SLN mapping was associated with a low incidence of postoperative lower extremity lymphedema in this single-arm cohort. These findings suggest that vNOTES-assisted SLN mapping may represent a feasible minimally invasive approach for nodal assessment in selected patients with endometrial cancer. Prospective comparative studies are required to confirm these findings and to evaluate long-term oncologic and lymphatic outcomes.

## Simple Summary

This study evaluates the use of a minimally invasive surgical technique, vNOTES, combined with sentinel lymph node mapping in patients with endometrial cancer. The results suggest that this approach is feasible and associated with a low rate of patient-reported lower extremity lymphedema. These findings highlight the potential of vNOTES to reduce surgical morbidity while maintaining effective nodal assessment.

## 1. Introduction

Endometrial cancer is the most common gynecologic malignancy in developed countries, with an estimated annual incidence of approximately 60,000 cases in the United States alone. It remains a major contributor to cancer-related morbidity and mortality among women worldwide [1,2]. Accurate surgical staging plays a pivotal role in guiding adjuvant therapy and optimizing oncologic outcomes. While systematic pelvic and para-aortic lymphadenectomy has traditionally been employed for this purpose, it carries a substantial risk of surgical morbidity [3,4].

Sentinel lymph node (SLN) mapping has emerged as a less invasive alternative for nodal assessment and has been increasingly incorporated into the surgical staging of endometrial cancer [5,6]. At the same time, advances in minimally invasive surgery have expanded the available operative approaches for endometrial cancer. Among these, vaginal natural orifice transluminal endoscopic surgery (vNOTES) has gained attention as a novel technique that combines endoscopic visualization with transvaginal access, allowing entry into the pelvic retroperitoneum [7]. This approach may facilitate SLN mapping and retroperitoneal dissection while also offering potential perioperative advantages such as reduced postoperative pain, faster recovery, shorter hospital stay, and improved cosmetic results [7,8].

One of the most significant complications following lymph node dissection is lower extremity lymphedema (LEL)—a chronic, often debilitating condition marked by the accumulation of lymphatic fluid in soft tissues [9,10]. LEL can severely impact quality of life through persistent swelling, discomfort, and functional impairment. Reported incidence rates following pelvic lymphadenectomy range from 20% to 40%, with risk factors including the extent of dissection, high body mass index, and adjuvant radiotherapy [11,12]. Despite its prevalence, lymphedema remains underdiagnosed, in part due to the absence of standardized diagnostic protocols and heterogeneous clinical presentations [9,11,12,13].

Against this backdrop, our study aimed to evaluate postoperative outcomes of vNOTES hysterectomy with bilateral salpingo-oophorectomy (BSO) and retroperitoneal SLN mapping using methylene blue dye in patients with newly diagnosed endometrial cancer. Our primary focus was the occurrence of postoperative lower extremity lymphedema, while also describing the surgical implementation of this approach in routine practice. Rather than assessing the diagnostic accuracy of SLN biopsy or comparing vNOTES with other surgical techniques, this study was designed as a retrospective cohort evaluating early lymphatic morbidity in a single-arm clinical series. To our knowledge, this is among the first studies to specifically report lower extremity lymphedema outcomes after vNOTES-assisted SLN mapping in this setting [14,15].

## 2. Materials and Methods

This retrospective cohort study was conducted at Gazi Yasargil Training and Research Hospital (Diyarbakır, Turkey) and approved by the institutional ethics committee (Approval No. 09-004-517). Data were collected from consecutive patients who underwent vNOTES hysterectomy with SLN mapping for newly diagnosed endometrial cancer during the study period (January 2022–January 2023). Eligible patients provided written informed consent. Inclusion criteria comprised a histologically confirmed diagnosis of endometrioid endometrial cancer, absence of synchronous malignancies or prior neoadjuvant therapy, and no radiological or metabolic evidence of lymph node metastasis on preoperative PET-CT imaging. All patients underwent vaginal natural orifice transluminal endoscopic surgery (vNOTES) hysterectomy with bilateral salpingo-oophorectomy (BSO) and sentinel lymph node (SLN) mapping using methylene blue dye. Patients with a prior diagnosis of lower extremity lymphedema, incomplete medical records, lymphedema due to disease recurrence, or contraindications to vNOTES or methylene blue were excluded from analysis.

All surgical procedures adhered to the standardized protocol established by Memorial Sloan Kettering Cancer Center. Methylene blue dye (1.25 mg/mL, 2–4 mL total) was injected into the cervix at the 3 and 9 o’clock positions. Retroperitoneal access was obtained via a 2–3 cm incision in the lateral vaginal fornix, through which a 7-cm GelPoint V-Path transvaginal access platform (Applied Medical, Rancho Santa Margarita, CA, USA) was inserted. CO_2_ insufflation was maintained at 12 mmHg. Dissection was performed in a caudal-to-cranial direction, identifying stained lymphatic channels and excising the SLNs transvaginally. Suspicious or bulky lymph nodes were also removed when necessary. If bilateral SLNs could not be visualized, side-specific or complete pelvic lymphadenectomy was performed. The procedure utilized a 30-degree laparoscope and bipolar and sealing instruments, with identification of key anatomical landmarks including the ureter, iliac vessels, and obturator nerve.

Sentinel lymph node mapping outcomes were recorded for all procedures. The overall SLN detection rate, bilateral detection rate, unilateral detection rate, and mapping failure rate were calculated. Bilateral detection was defined as identification of at least one SLN on each pelvic side, whereas unilateral detection referred to SLN identification on only one side. Cases in which no SLN was identified were considered mapping failures. In accordance with the surgical algorithm, side-specific pelvic lymphadenectomy was performed when SLN mapping was unsuccessful on one side, and complete pelvic lymphadenectomy was performed when mapping failed bilaterally.

Postoperative staging followed the 2008 FIGO classification. Adjuvant radiotherapy was administered to patients with stage II, IIIA, or IIIB disease, consisting of whole pelvic external beam radiation (50.4 Gy over 5–6 weeks) with or without vaginal brachytherapy. Chemotherapy was indicated for serous or clear cell histologies or advanced-stage disease, comprising six cycles of carboplatin and paclitaxel, with or without epirubicin, depending on individual patient performance status.

Lower extremity lymphedema (LEL) was evaluated using the Turkish-validated version of the 7th edition Gynecologic Cancer Lymphedema Questionnaire. A cumulative score above 5 was considered diagnostic for lymphedema. The questionnaire has been shown to have high sensitivity and specificity in both obese and non-obese populations. The questionnaire was administered as part of routine postoperative follow-up in our institution, and the subsequent validation study formally confirmed the reliability of the Turkish version. Patients completed the questionnaire at 6 and 12 months postoperatively as part of routine follow-up. Additionally, physical examinations were performed by both a gynecologic oncologist and a physiatrist to confirm patient-reported symptoms. Patients who developed edema due to disease recurrence were excluded from the lymphedema analysis.

This methodology allowed for consistent surgical technique, standardized outcome assessment, and ethical adherence throughout the study period.

### Statistical Analysis

Statistical data analysis was performed in the Statistical Package for Social Sciences (SPSS), version 25 (IBM Corp., Armonk, NY, USA). Results are expressed as mean ± standard deviation or median and minimum–maximum levels according to the distributional characteristics of the data. The normality of the numerical variables was examined using visual (histograms and probability plots), Kolmogorov–Smirnov and Shapiro–Wilk tests. Normally distributed numerical variables were presented as mean and standard deviation, while non-normally distributed numerical variables were presented as median and interquartile range. Chi-square or Fisher’s exact tests were used for categorical variables in comparisons between independent groups. A *p*-value < 0.05 was considered statistically significant.

No formal power analysis was performed, as this was a single-arm, retrospective study primarily aimed at reporting the incidence of lower extremity lymphedema rather than testing specific hypotheses. Given the extremely low event rate (*n* = 1), multivariate logistic regression analysis could not be conducted due to perfect separation, and statistical comparisons should be interpreted as descriptive and exploratory.

## 3. Results

A total of 113 patients who underwent vNOTES surgery with sentinel lymph node mapping for the management of newly diagnosed endometrial cancer were included in the present study. The mean age was 55.0 ± 10.5 years (range: 34–78), and the mean body mass index (BMI) was 30.94 ± 2.54 kg/m^2^. The mean number of lymph nodes removed per patient was 3.87 ± 3.47. In patients with successful SLN mapping, the number of retrieved nodes was consistent with typical SLN yields, whereas higher counts were observed in patients requiring side-specific or complete lymphadenectomy following mapping failure. Sentinel lymph node mapping was successful in 102 of 113 patients, yielding an overall detection rate of 90.2%. Bilateral SLN detection was achieved in 90 patients (79.6%), while unilateral detection occurred in 12 patients (10.6%). Mapping failure was observed in 11 patients (9.7%). In cases with unsuccessful mapping on one side, side-specific pelvic lymphadenectomy was performed according to the surgical algorithm.

Histopathological evaluation revealed that 75.5% of patients had endometrioid adenocarcinoma, followed by endometrial intraepithelial neoplasia (EIN; 17.3%), serous carcinoma (5.1%), clear cell carcinoma (1.0%), and mixed histology (1.0%). Tumor grading showed that 57.1% were grade 1, 31.6% were grade 2, and 11.2% were grade 3. The most frequent stages were IA (25.5%) and EIN (17.3%). The detailed demographic and clinical characteristics are summarized in Table 1.

Adjuvant radiotherapy was administered to patients with stage II–III disease according to institutional treatment protocols. Among patients with nodal metastasis (*n* = 10), 70% received pelvic external beam radiotherapy and 30% received vaginal brachytherapy. In patients requiring additional lymphadenectomy due to mapping failure (*n* = 11), radiotherapy was administered according to final pathological risk stratification.

Among patients with complete lymphedema assessment data (*n* = 98), postoperative lymphedema-related symptoms were uncommon (Table 2). Limb swelling was reported in 4.1% (*n* = 5), heaviness in 9.2% (*n* = 10), tightness in 1.0% (*n* = 1), and pain in 1.0% (*n* = 1) of patients. No clinical signs of inguinal lymphedema were detected during physical examination. Only one patient (1.0%) met the criteria for self-reported mild lymphedema based on the validated questionnaire.

Due to the extremely low number of patients meeting the diagnostic criteria for lymphedema, no multivariate analysis could be performed. Statistical comparisons between clinical variables and lymphedema-related symptoms should be interpreted as exploratory. Self-reported mild lymphedema was significantly associated with swelling (*p* = 0.041), tightness (*p* = 0.010), and pain (*p* = 0.010), as shown in Table 3.

## 4. Discussion

Our findings position vNOTES with sentinel lymph node (SLN) mapping as a promising advancement in endometrial cancer surgery, demonstrating a low rate of patient-reported postoperative lower extremity lymphedema symptoms (1.0%). This contrasts sharply with the historically reported rates of 20–40% following systematic lymphadenectomy [7,8], suggesting that this approach may be associated with low lymphatic morbidity in appropriately selected patients undergoing endometrial cancer surgery.

The 1.0% lymphedema rate observed in this retrospective cohort study is, to our knowledge, a low rate compared to previously reported data on endometrial cancer surgery. While SLN mapping alone has been consistently shown to reduce lymphatic morbidity [14,16,17,18], several factors may contribute to the additional benefit observed with vNOTES. Transvaginal retroperitoneal access may limit disruption of abdominal wall lymphatics, enhance precision through improved visualization, and reduce the extent of nodal excision due to higher SLN detection rates. These features may contribute to a surgical approach that provides adequate nodal assessment while maintaining low postoperative lymphatic morbidity [19,20,21,22]. Our results align with the findings of the SENTI-ENDO and FIRES trials, which confirmed the diagnostic accuracy and safety of SLN mapping in endometrial cancer [5,6]. Additionally, vNOTES has been associated with reduced postoperative pain, shorter hospital stays, and faster recovery compared to traditional laparoscopy [7,17,19,23]. The present study adds to this evidence by reporting a low incidence of lymphedema in patients undergoing vNOTES with SLN mapping.

The relatively higher mean number of retrieved lymph nodes in our cohort (3.87 ± 3.47), compared to prior studies, may be explained by the inclusion of patients who underwent side-specific or complete lymphadenectomy in cases of mapping failure [20,21] as well as the potential retrieval of nodal clusters rather than single sentinel nodes due to the surgical approach and dye-based mapping technique [19,22].

Notably, our study cohort had a mean BMI of 30.9 kg/m^2^, yet lymphedema incidence remained low, supporting the notion that minimally invasive techniques with limited nodal dissection may mitigate known risk factors such as obesity and extended lymphadenectomy [12,24,25,26,27]. Our use of the validated Gynecologic Cancer Lymphedema Questionnaire ensured consistent and culturally adapted symptom detection [24]. However, because lymphedema assessment relied primarily on patient-reported symptoms rather than objective volumetric measurements, the possibility that some reported lower extremity swelling could be related to non-lymphatic causes, such as chronic venous insufficiency or metabolic comorbidities, cannot be completely excluded. In contrast, prospective studies with baseline and objective measurements, such as Bjørnholt et al. [28], have reported higher rates of clinically relevant lymphedema (approximately~7%) following SLN mapping, suggesting that our findings likely reflect patient-reported symptoms rather than the true incidence of lymphedema.

However, several limitations must be acknowledged. The study’s retrospective design introduces potential selection bias. Only one patient in the cohort met the diagnostic criteria for lymphedema, precluding the feasibility of multivariate statistical modeling. While exploratory associations were examined, the findings should be interpreted descriptively and not as confirmatory evidence. Additionally, the lack of a comparator group (e.g., conventional laparoscopy or robotic-assisted SLN mapping) limits the ability to attribute the low incidence solely to the vNOTES technique. Furthermore, although adjuvant radiotherapy is a known risk factor for the development of lower extremity lymphedema, the small number of patients receiving postoperative radiation therapy in this cohort limited the ability to evaluate its potential impact on lymphedema outcomes.

Importantly, this study was not designed as a comparative analysis. Given that vNOTES with sentinel lymph node mapping is a relatively novel technique, our aim was to assess feasibility, safety, and early morbidity outcomes in a single-arm observational cohort. While comparisons with other surgical modalities may be informative, they were beyond the scope of this investigation. Future prospective studies including comparative arms will be essential to determine relative advantages in terms of lymphatic and oncologic outcomes.

Finally, no objective volumetric measurements (e.g., bioimpedance spectroscopy or limb circumference) were used, which may underestimate subclinical lymphedema. In addition, SLN mapping in the present study was performed using methylene blue dye, whereas indocyanine green fluorescence imaging is currently recommended in many guidelines due to its higher detection rates. Therefore, the use of methylene blue alone may represent a methodological limitation of the present study. Despite these limitations, our study is among the first to focus on lymphedema outcomes following vNOTES-assisted SLN mapping and highlights the need for future prospective, multicenter studies to validate these findings and explore long-term morbidity outcomes.

## 5. Conclusions

In conclusion, vNOTES hysterectomy combined with retroperitoneal sentinel lymph node mapping appears to be a feasible and safe surgical approach for the management of early-stage endometrial cancer. In our cohort, this technique was associated with a remarkably low incidence of postoperative lower extremity lymphedema. These findings suggest that vNOTES-assisted SLN mapping may be associated with low lymphatic morbidity in appropriately selected patients. By minimizing surgical morbidity while preserving nodal assessment, this approach may represent a valuable minimally invasive option in endometrial cancer surgery. Given the single-arm design of the present study, no conclusions can be drawn regarding superiority over conventional laparoscopic or open techniques. Furthermore, the present study was not designed to evaluate long-term oncologic outcomes, and SLN mapping was performed using methylene blue dye rather than indocyanine green fluorescence imaging. Prospective, comparative trials are needed to validate these results and to establish standardized protocols for integrating vNOTES into gynecologic oncology practice.

## Figures and Tables

**Table 1:** Demographic and Clinical Characteristics of the Study Population (data are presented as mean ± SD or %).

Variable	Value
Number of patients	113
Mean age (years)	55.0 ± 10.5 (range 34–78)
Mean BMI (kg/m^2^)	30.94 ± 2.54
Mean number of lymph nodes removed	3.87 ± 3.47
Nulliparity	7 (6.2%)
Previous vaginal delivery	88 (77.9%)
Previous cesarean section	20 (17.7%)
Previous abdominal surgery	4 (3.5%)
Diabetes mellitus	19 (16.8%)
Hypertension	30 (26.5%)
Histology	Endometrioid (75.5%), EIN (17.3%), Serous (5.1%), Clear cell (1.0%), Mixed (1.0%)
Tumor grade	Grade 1 (57.1%), Grade 2 (31.6%), Grade 3 (11.2%)
Most frequent stages	IA (25.5%), EIN (17.3%)

Statistical Method: Descriptive statistics were used to summarize demographic and clinical characteristics. Continuous variables were presented as mean ± standard deviation (SD) after assessing normality with the Kolmogorov–Smirnov and Shapiro–Wilk tests.

**Table 2:** Incidence of Postoperative Lymphedema-Related Symptoms. Percentages are calculated based on the total cohort (*n* = 113). Calculations based on SLN-detected subgroups would yield slightly different proportions, and when restricted to patients with successful SLN mapping (*n* = 102), the proportions were similar (e.g., limb swelling: 4.9%).

Symptom	Number of Patients	Percentage (%)
Limb swelling	5	4.4%
Sense of heaviness	10	8.8%
Tightness sensation	1	0.9%
Pain sensation	1	1.0%
Inguinal lymphedema (clinical)	0	0%
Self-reported mild lymphedema	1	0.9%

Statistical Method: Frequency and percentage distributions were used to describe the incidence of self-reported and clinically observed lymphedema-related symptoms.

**Table 3:** Association Between Self-Reported Mild Lymphedema and Symptom Profile (significance set at *p* < 0.05).

Symptom	*p*-Value
Swelling	0.041
Tightness	0.010
Pain	0.010

Statistical Method: Associations between self-reported mild lymphedema and individual symptoms were analyzed using Fisher’s exact test due to small sample sizes. A *p*-value of <0.05 was considered statistically significant.

## Data Availability

The data presented in this study are available on request from the corresponding author due to privacy and ethical restrictions.
